# Opioid Analgesic Use After an Acute Pain Visit: Evidence from a Urolithiasis Patient Cohort

**DOI:** 10.5811/westjem.2022.8.56679

**Published:** 2022-10-23

**Authors:** Anna E. Wentz, Ralph C. Wang, Brandon D.L. Marshall, Theresa I. Shireman, Tao Liu, Roland C. Merchant

**Affiliations:** * Brown University School of Public Health, Department of Epidemiology, Providence, Rhode Island; † University of California, San Francisco, Department of Emergency Medicine, San Francisco, California; ‡ Brown University School of Public Health, Health Services Policy & Practice, Providence, Rhode Island; § Brown University School of Public Health, Data & Statistics Core of Brown Alcohol Research Center on HIV (ARCH), Providence, Rhode Island; ¶ Harvard Medical School, Brigham and Women’s Hospital Department of Emergency Medicine, Boston, Massachusetts

## Abstract

**Introduction:**

Urolithiasis causes severe acute pain and is commonly treated with opioid analgesics in the emergency department (ED). We examined opioid analgesic use after episodes of acute pain.

**Methods:**

Using data from a longitudinal trial of ED patients with urolithiasis, we constructed multivariable models to estimate the adjusted probability of opioid analgesic use 3, 7, 30, and 90 days after ED discharge. We used multiple imputation to account for missing data and weighting to account for the propensity to be prescribed an opioid analgesic at ED discharge. We used weighted multivariable regression to compare longitudinal opioid analgesic use for those prescribed vs not prescribed an opioid analgesic at discharge, stratified by reported pain at ED discharge.

**Results:**

Among 892 adult ED patients with urolithiasis, 79% were prescribed an opioid analgesic at ED discharge. Regardless of reporting pain at ED discharge, those who were prescribed an opioid analgesic were significantly more likely to report using it one, three, and seven days after the visit in weighted multivariable analysis. Among those who were not prescribed an opioid analgesic, an estimated 21% (not reporting pain at ED discharge) and 30% (reporting pain at discharge) reported opioid analgesic use at day three. Among those prescribed an opioid analgesic, 49% (no pain at discharge) and 52% (with pain at discharge) reported using an opioid analgesic at day three.

**Conclusion:**

Urolithiasis patients who received an opioid analgesic at ED discharge were more likely to continue using an opioid analgesic than those who did not receive a prescription at the initial visit, despite the time-limited nature of urolithiasis.

## INTRODUCTION

Urolithiasis is an acute painful condition with increasing prevalence globally.^1^–^4^ The pain caused by urolithiasis is commonly referred to as renal colic, and usually resolves when the stone passes into the bladder within a few hours or up to a few days.^5^,^6^ On average, most stones are fully expelled within about two weeks, with some variation depending on the size and location of the stone.^7^ Current standard of care for managing acute urolithiasis pain in the emergency department (ED) is to treat pain with non-steroidal anti-inflammatory drugs (NSAID), unless they are contraindicated for the patient or unless NSAIDs are not providing sufficient pain relief. 8–10 However, in practice, opioid analgesics are often used to manage pain for patients with urolithiasis.^11^,^12^ Older clinical guidance often suggests administering opioids and NSAIDs together in the ED^5^,^13^ and to “titrate up the analgesic ladder according to pain.”^14^ Despite these recommendations for pain relief during the ED visit, guidance on pain management after the ED visit is not well established but can include oral NSAIDs with or without opioid analgesics.^15^

Overall opioid prescribing in the United States has decreased since 2012 as efforts have been underway to limit access to these medications in response to the opioid epidemic.^16^ However, decreases in prescribing for severe acute conditions, such as urolithiasis, have been relatively small in comparison to the overall decrease in prescribing.^17^–^19^ While opioid overdose deaths had previously begun to decrease, they became higher than ever during the COVID-19 pandemic.^20^ There is evidence that even a one-time prescription for an opioid analgesic may result in long-term opioid use after a dental procedure,^21^ pregnancy,^22^ and ED visits for back pain^23^ or ankle sprains,^24^ but this has not been studied for patients with urolithiasis, a condition characterized by severe acute pain. Now may be a critical time to revisit opioid analgesic prescribing when discharging ED patients with renal colic.

Our objective was to determine whether ED patients with urolithiasis, a time-limited, acute pain condition, who were discharged with an opioid analgesic prescription were more likely to report opioid analgesic use after the ED visit than patients who did not receive a prescription at the end of the ED visit. We specifically aimed to compare prescription opioid analgesic use after the ED visit according to two groups of patients: those who were still in pain at the time of ED discharge vs those who were no longer reporting pain. Using existing data from a randomized controlled trial,^25^ in this investigation we examined the outcome of prescription opioid analgesic use after ED discharge by comparing four cohorts of patients based on whether they received an opioid analgesic prescription and whether they reported any or no pain at the time of ED discharge. We hypothesized that ED patients who received an opioid analgesic prescription at ED discharge would use this medication, regardless of reporting pain, longer than those who were not prescribed them.

## METHODS

We analyzed data collected as part of a parent randomized controlled trial (the STONE [Study of Tomography of Nephrolithiasis Evaluation] trial; R01HS019312) that was initially designed to compare diagnostic techniques for urolithiasis.^25^ Detailed methods are reported elsewhere.^26^ From 2011–2013 in the STONE trial, trained research coordinators invited adult patients 18–75 years old with suspected urolithiasis from 15 EDs across the US to participate in the trial. Participants were randomly assigned to one of three diagnostic techniques (point-of-care ultrasound in the ED, ultrasound in radiology, or computed tomography), and contacted for follow-up via phone at 3, 7, 30, and 90 days after ED discharge. The Brown University Institutional Review Board determined this secondary analysis of deidentified data did not involve human subjects. Funding for this project was provided by the National Institutes of Health [F31DK124898]. None of the funding sources for this project played any role in the conduct of the study, study design, analysis, manuscript preparation, or decision to submit the manuscript for publication.

**Population Health Research Capsule**

What do we already know about this issue?

*Over the past decade opioid analgesic prescribing has declined in the US overall, but prescribing for urolithiasis, a severe acute pain, has been slow to decrease*.

What was the research question?

*Does an opioid analgesic prescription at ED discharge affect continued use after an ED visit for urolithiasis?*

What was the major finding of the study?

*Urolithiasis patients discharged with a prescription were 1.8 to 3.6 times more likely be using opioids at 3 and 7 days than those discharged without an opioid prescription*.

How does this improve population health?

*Although urolithiasis is acute and expected to resolve quickly, further limiting prescription of opioid analgesics might prevent their prolonged use after urolithiasis*.

### Study Population

**Figure 1:**
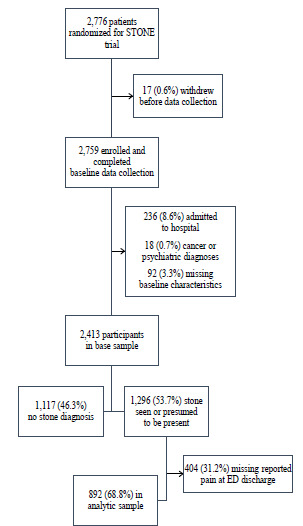
Enrollment and retention in the analytic sample from the STONE trial. *STONE*, Study of Tomography of Nephrolithiasis Evaluation; *ED*, emergency department.

The parent trial enrolled 2,759 adult ED patients with suspected urolithiasis (acute renal colic based on clinical presentation) for whom treating physicians ordered diagnostic imaging. The parent trial invited ED patients with suspected urolithiasis who were between 18–75 years of age, not pregnant or obese, and had no history of nephrectomy, renal transplant, or dialysis to participate in the study.^26^ The sample for this secondary analysis included participants with complete information on baseline characteristics and reported pain at the time of discharge from the ED. We excluded participants who were admitted to the hospital and those receiving a psychiatric or cancer diagnosis at the ED visit (Figure 1). This secondary analysis focused on the subpopulation of participants diagnosed with urolithiasis (n = 1,296). For a sensitivity analysis (described below), we used the larger population of patients with suspected urolithiasis (n = 2,413). We performed the sensitivity analysis to explore whether the findings of longitudinal opioid analgesic use were consistent or there were differences in use between the population diagnosed with urolithiasis as compared to those whose diagnosis was less specific.

### Exposure

For our primary analysis, we defined four cohorts based on two exposure variables at the time of ED discharge: receipt of an opioid analgesic prescription (recorded from medical records); and pain (reported on a 0–10 scale and dichotomized to any [≥1] or no [0] reported pain). The four cohorts were as follows: 1) reported no pain at ED discharge and did not receive an opioid analgesic prescription; 2) reported pain at discharge but did not receive an opioid analgesic prescription; 3) reported no pain at discharge but received an opioid analgesic prescription; and 4) reported pain and received an opioid analgesic prescription at discharge.

### Outcome

At each follow-up (3, 7, 30, and 90 days post-ED visit), trained research coordinators asked participants whether they were currently taking an opioid analgesic to treat the pain due to the original condition that brought them to the ED when they enrolled in the trial.^26^ Our primary goal was to compare post-ED opioid analgesic use at follow-up among the four cohorts of participants described previously.

### Analysis

We described characteristics of the study sample, providing the count and percentage for categorical variables and median and interquartile range IQR) for continuous variables. Because receipt of an opioid analgesic prescription at ED discharge was not randomly assigned, we enacted statistical measures to reduce confounding by factors that might have influenced which patients received opioid analgesic prescriptions. Using the population of patients with suspected urolithiasis (n = 2,413), we used inverse propensity score weighting^27^ to adjust for the probability of receiving an opioid analgesic prescription. The intended result of the adjustment was to simulate random assignment to receipt of an opioid analgesic prescription by attempting to account for factors that might have contributed to patients receiving vs not receiving the prescription.

For this adjustment we estimated the conditional probability of receiving an opioid prescription at ED discharge, given covariates previously identified to predict that probability in this population: urolithiasis diagnosis; gender; age; education level; race/ethnicity; self-rated health; health insurance status; pain level at ED arrival; duration of pain prior to arrival; calendar time; and presence of a prescription drug monitoring program (PDMP) in the state at the time of the visit.^11^ These estimates produced a propensity score for each participant. Per recommended practice,^28^ we used the propensity score to weight the data according to the inverse of the probability of receiving an opioid analgesic prescription at ED discharge.

Next, we imputed values to substitute for missing data on opioid analgesic use at follow-ups with chained equations^29^ using patient and visit characteristics shown in published research to be associated with persistent opioid analgesic use. We used predictors of opioid use after an ED visit (opioid analgesic administration during the visit, gender, age, urolithiasis diagnosis, education, race/ethnicity, self-rated health, pain at ED arrival, duration of pain prior to ED arrival, calendar time, and presence of an online PDMP in the state at the time of the visit) to predict the missing values for reported opioid use at day 3.^30^ Our imputation model then used the same predictors with the addition of opioid use at day 3 to predict missing values for opioid use at day 7. The chained equations repeated this pattern for day 30 and then day 90. We created 25 imputed datasets and performed pooled statistical inference.^31^

In the multiple imputed data sets, we first focused on the subpopulation of urolithiasis-diagnosed participants not missing reported pain at ED discharge (n = 892). In this subpopulation we used generalized estimating equations to account for within-subject correlation due to repeated follow-ups, and we constructed multivariable generalized linear models with a logit link to estimate the odds of using an opioid analgesic at follow-up for each of the four cohorts. We created a dummy variable to index each follow-up to capture any non-linear pattern of using an opioid analgesic after ED discharge and allowed the responses to change over time for participants who were prescribed vs not prescribed an opioid analgesic at discharge by including an applicable interaction term. The inverse probability weights were then used to adjust for factors that influenced which patients received an opioid analgesic prescription at ED discharge.

This final model produced estimated odds ratios (OR) of using an opioid analgesic at each follow-up visit comparing those with and without an opioid analgesic prescription at ED discharge, stratified by whether or not the participant reported any pain at the end of the ED visit. Additionally, for each of these four cohorts, stratified by opioid analgesic prescription and overall we used the multivariable model to estimate the adjusted percentage with a 95% confidence interval (CI) of participants who reported using an opioid analgesic at each follow-up.

In a sensitivity analysis, we repeated the analysis in the larger subpopulation of participants with suspected urolithiasis and complete data on pain at ED discharge (n = 1,580).

## RESULTS

Of the 2,759 participants enrolled in the STONE trial, we excluded 12.5% (n = 346) who were admitted to the hospital, had a cancer or psychiatric diagnosis, or were missing baseline characteristics (Figure 1). Of the remaining 2,413 participants, just over half (1,296) were diagnosed with urolithiasis, and of those, 892 (68.8%) reported whether they were experiencing pain at the end of the ED visit and thus were included in our main analysis (Figure 1). In this analytic sample, there was a very high level of pain reported at ED arrival, with 57.4% (n = 512) of participants rating their initial pain at a 9 or 10 on the 0–10 scale. By the time these patients were discharged from the ED, 68.4% (n = 610) reported experiencing any pain (1–10). The pain scores reported at the end of the ED visit were much lower than at arrival, with a median (IQR) of 9 (8–10) at arrival and 2 (0–4) at discharge. Nearly 80% of this group received a prescription for an opioid analgesic at the time of ED discharge (Table 1). Seventy-five percent (n = 2,692) of follow-up observations had non-missing data for reported opioid analgesic use.

In multivariable analysis using inverse probability weighting to adjust for the probability of receiving an opioid analgesic prescription at ED discharge, there were significant differences in opioid analgesic use at follow-up between cohorts. Regardless of whether participants had pain at the end of the ED visit, those who were prescribed an opioid analgesic were more likely to report using one, three and seven days after the visit (Table 2). For example, in the cohorts not reporting pain at ED discharge, those receiving an opioid analgesic prescription had OR = 3.63 (95% CI 1.87–7.07) greater odds of using an opioid analgesic at day 3 than those not receiving a prescription (Table 2, first column).

**Figure 2:**
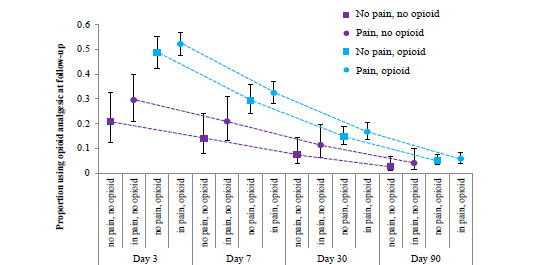
Adjusted proportion (95% confidence interval (in whiskers) of participants reporting pain at follow-up visits by cohort estimated by multivariable model adjusted for propensity to receive an opioid analgesic prescription, stratified by pain reported and prescription of opioid analgesic at emergency department discharge. Sample includes 3,568 follow-up observations for 892 adults with urolithiasis seen at one of 15 emergency departments in the STONE trial. *STONE*, Study of Tomography of Nephrolithiasis Evaluation.

Figure 2 shows the estimated percentage of participants using an opioid analgesic at each follow-up from the multivariable models. Here we see that the differences in the proportion using an opioid analgesic become smaller by day 7. However, regardless of whether a participant reported pain at the end of the ED visit, those who received an opioid analgesic prescription at ED discharge were more likely than those not receiving a prescription to report using an opioid analgesic three days after the visit (Figure 2). For the two cohorts *not prescribed* an opioid at discharge, an estimated 21% of those with no pain and 30% of those with pain reported using an opioid analgesic at the three-day follow-up interview. In contrast, for the cohorts that were prescribed an opioid analgesic, 49% of those not reporting pain at discharge and 52% of those in pain at discharge reported using an opioid analgesic 3 days after the visit (Figure 2).

When the cohorts were combined to compare those receiving vs not receiving an opioid analgesic prescription regardless of pain at ED discharge, 31.6% (27.8–35.6%) of those receiving an opioid analgesic prescription at discharge reported using an opioid seven days after the visit, while only 18.0% (12.0–26.1%) of those without an opioid analgesic prescription at ED discharge reported using one. At day 30 and day 90, the difference between groups was not statistically significant. Overall, in the total population of acute renal colic patients in this study, an estimated 12.5% (9.4–16.4%) reported using an opioid analgesic to treat that pain 30 days later, and 4.3% (2.6–7.1%) were still doing so 90 days after the initial ED visit.

In the sensitivity analysis including the larger population of participants with suspected urolithiasis, the association between receiving an opioid analgesic prescription at ED discharge and using that prescription was slightly stronger for both those reporting pain at the time of ED discharge and no longer experiencing pain, especially at day 3 (Appendix Table 3). Appendix Figure 1 shows the adjusted probability of using an opioid analgesic at each follow-up visit in this larger sample. This sensitivity analysis followed similar relative trends as our main analysis, but the overall probability of using an opioid analgesic was higher at each time point in the larger sample and the CIs more precise.

## DISCUSSION

Thirty days after an ED visit for urolithiasis, 7–17% of patients in our study sample reported using an opioid analgesic to treat the pain that prompted their visit (Figure 2). If prescribed an opioid analgesic, patients were more likely to continue using an opioid analgesic after the visit than those not receiving a prescription. Of greater importance, the association between receiving an initial opioid analgesic prescription and post-ED prescription opioid analgesic usage remained higher among those who did not report pain at ED discharge than those who did. The differences we observed between those receiving an opioid analgesic prescription vs not diminished over time, but we did observe a trend for more usage in the group receiving a prescription.

In our sensitivity analysis including all participants with suspected urolithiasis, we observed similar trends (Appendix Table 3), but more precise estimates and a higher probability of reported opioid analgesic use was higher at all follow-ups in the larger population (Appendix Figure 1). Higher probability of opioid analgesic use in the population of patients with suspected urolithiasis was as expected, due to the time-limited nature of urolithiasis (once a stone passes, the pain subsides). Patients with other diagnoses are likely to continue experiencing pain for longer and, therefore, continue using opioid analgesics as well, especially if prescribed.

Our study is not the first to find ongoing opioid analgesic use after acute pain more likely for patients prescribed an opioid analgesic. In one study of ED patients with a broad array of acute painful complaints, 17% of patients who filled their opioid analgesic prescription continued receiving it one year later.^32^ A study of motor vehicle collisions found that, six weeks after the collision, participants prescribed opioid analgesics were more likely to report using prescription opioids than those prescribed NSAIDs.^33^ Another study of ED patients with acute pain who were discharged with an opioid prescription found that those who used the prescription during the first two weeks after discharge were 3.8 times as likely to use opioids three months later than those who did not use opioids during the first two weeks after the visit.^34^

A recent retrospective study shared a similar concern for patients with acute urolithiasis.^12^ Of 271 patients at a single hospital with urolithiasis in 2017–2020, 66% received an opioid prescription at the initial visit. In our sample of urolithiasis patients with ED visits occurring from 2011–2013, 78% of patients received an opioid analgesic prescription at discharge. The difference in the proportion of patients receiving an opioid analgesic prescription between this study and that in Cotta et al^12^ could be due to several factors: variation in prescribing between hospitals (within hospitals in this study 52–95% of visits received an opioid analgesic prescription); our sample of ED visits (Cotta et al noted that patients with an initial visit to the ED were more likely to receive a prescription than if the initial visit was at an urgent care or another clinic type)^12^; and finally, a possible decrease in prescribing over time, although this change has been found to be minimal for urolithiasis compared to other contexts.^17^–^19^ Consistent with our results, the retrospective study found that those who received an opioid analgesic prescription at their initial visit were more likely to require a refill during the acute stone episode than those who did not receive an opioid prescription at the initial visit.

## LIMITATIONS

This analysis is subject to several limitations. As with all longitudinal studies, this investigation had missing data on reported pain at ED discharge and opioid analgesic use at follow-ups. A smaller number of baseline characteristic data was missing; however, excluding this small portion of participants (3.3%) should not meaningfully have changed our results. Unfortunately, nearly one third of the sample was missing reported pain at ED discharge. We believe that pain at the end of the visit was important information needed to answer the research question about whether participants who receive an opioid analgesic prescription at discharge were more likely to continue using an opioid analgesic after the visit, and that 892 participants was sufficient to build our multivariable models. The point when patients were leaving the ED proved to be a difficult time for study staff to obtain information from patients, so missingness at this time point might not be related to the exposure or outcome in this study. We chose to omit those participants without pain data at ED discharge from this analysis and not attempt to impute reported pain at discharge in addition to our outcome.

One quarter of follow-up observations were missing reported opioid analgesic use, the outcome for this analysis. Omitting this missing information could result in biased estimates, as this data was not missing completely at random. To overcome that limitation, rather than assume the missing data was similar to that observed, we elected to use multiple imputation with chained equations to fill in values for those missing observations,^29^ and we performed pooled statistical inference to produce a final estimate from the 25 imputed datasets.^31^

Another potential limitation of this study is that overall opioid analgesic prescribing has changed since the data was collected in 2011–2013. However, opioid analgesic prescribing for acute painful conditions such as urolithiasis has decreased very slowly compared to overall prescribing.^17^–^19^ In this patient population, 79.6% (n = 710) of patients with urolithiasis received an opioid analgesic prescription at ED discharge. This is higher than national estimates for 2012–2013, when 56.9% of urolithiasis patients received an opioid analgesic prescription at ED discharge, and for 2016–2017, when the national estimate was 49.2%.^17^ For the years 2013–2018, other individual EDs reported decreases from 70% to 52%^19^ and from 81% to 59%.^18^ Had the present investigation been repeated in 2018, we would expect to see fewer than 79.6% of urolithiasis patients receive an opioid analgesic prescription at ED discharge, likely close to the 59% observed in the Kominsky et al study.^18^ However, we do not have reason to expect the relationship between opioid analgesic prescribing and use to change over time. Hence, we believe these results remain applicable to current ED practice.

Finally, the parent study did not collect information on exposure to opioid analgesics prior to the ED visit, opioid use disorder, or existing chronic pain or mental health conditions. These factors could have influenced both physician prescribing and opioid analgesic use at follow-up, and thus could have affected our results. Cotta et al were able to control for an existing prescription and still found that patients who received an opioid analgesic prescription at the initial visit for urolithiasis were more likely to refill a prescription than those not receiving a prescription at the initial visit.^12^ Other studies including only opioid-naïve patients have found continued opioid analgesic use more likely for those prescribed an opioid analgesic at the initial acute pain ED visit than those not prescribed an opioid analgesic for ankle sprains,^24^ back pain,^23^ and acute pain in general.^32^

It is not well established whether having opioid use disorder means a patient is more, perhaps due to requesting the medication, or less, if the physician is aware of the patient history, likely to receive an opioid analgesic prescription. The parent study also did not track how many pills of opioid analgesics were prescribed or refills received, which would both be confounding variables. Related to refills, it is not known whether participants who reported using an opioid analgesic 90 days after the initial ED visit received a refill, a new prescription, or were using remaining pills from the original prescription.

## CONCLUSION

In this secondary analysis of a longitudinal study of acute urolithiasis ED patients, one eighth of our sample reported using an opioid analgesic 30 days after the visit. We found that those prescribed an opioid analgesic at ED discharge were more likely to report using opioid analgesics a week after the visit than those who did not receive an opioid prescription. Of great importance, opioid analgesic use continued for those who had reported that their pain was relieved at the end of the ED visit. The most current practice guidelines for managing pain due to urolithiasis suggest reserving opioid analgesics as a last resort. Yet opioid analgesics are still often prescribed for urolithiasis, and our study findings show they are prescribed at ED discharge even when a patient’s pain has resolved by the end of the visit. Especially given evidence that NSAIDs are more effective at pain reduction for urolithiasis, further limiting prescription of opioid analgesics at ED discharge might prevent their prolonged use.

## Supplementary Information



## Figures and Tables

**Table 1:** Clinical and demographic characteristics of 892 adults with urolithiasis seen at one of 15 US emergency departments, STONE trial.

Descriptive characteristic	n (%)
Gender	
Female	343 (38.5)
Male	549 (61.6)
Age (years), median (IQR)	38 (28–48.5)
Years of formal education	
High school graduate or less	406 (45.5)
Some post-high school education	232 (26.0)
College graduate	254 (28.5)
Race/ethnicity	
Black	139 (15.6)
Hispanic	228 (25.6)
Non-Hispanic White	437 (49.0)
Mixed or other race	88 (9.9)
Has healthcare insurance	651 (73.0)
Pain at ED arrival	
Low (0–3)	43 (4.8)
Medium (4–8)	337 (37.8)
High (9–10)	512 (57.4)
Duration of pain before arrival to ED	
1 to 2 hours	233 (26.1)
3 to 6 hours	228 (25.6)
7 to 12 hours	109 (12.2)
13 to 24 hours	72 (8.1)
25 to 48 hours	62 (7.0)
> 48 hours	188 (21.1)
Self-rated health	
Excellent	162 (18.2)
Very good	256 (28.7)
Good	316 (35.4)
Fair	132 (14.8)
Poor	26 (2.9)
ED visit in state with PDMP online access	466 (52.2)
Opioid analgesic administered during ED visit	661 (74.1)
Opioid analgesic prescription at ED discharge	710 (79.6)
Reported any pain at ED discharge	610 (68.4)

*STONE*, Study of Tomography of Nephrolithiasis Evaluation; *IQR*, interquartile range; *ED*, emergency department; *PDMP*, prescription drug monitoring program; *US*, United States.

**Table 2:** Odds ratios of using an opioid analgesic at each post-emergency department (ED) visit follow-up by reported pain at the end of the ED visit and receipt of prescription opioid analgesic from the ED. Estimates from multivariable model weighted for propensity to receive an opioid analgesic prescription at ED discharge and using multiple imputations with chained equations to account for missing outcome data at follow-ups. Sample includes 3,568 follow-up observations for 892 adults with urolithiasis seen at one of 15 EDs in the STONE trial.

Outcome	No pain at discharge	Reported pain at discharge
Exposure group	OR (95% CI)	OR (95% CI)
Opioid analgesic use at day 3		
No opioid prescribed at ED discharge	ref	ref
Opioid prescribed at discharge	3.63 (1.87–7.07)	2.61 (1.60–4.27)
Opioid analgesic use at day 7		
No opioid prescribed at ED discharge	ref	ref
Opioid prescribed at discharge	2.53 (1.26–5.09)	1.81 (1.03–3.22)
Opioid analgesic use at day 30		
No opioid prescribed at ED discharge	ref	ref
Opioid prescribed at discharge	2.18 (1.00–4.78)	1.57 (0.79–3.12)
Opioid analgesic use at day 90		
No opioid prescribed at ED discharge	ref	ref
Opioid prescribed at discharge	2.01 (0.70–5.80)	1.44 (0.53–3.92)

*ED*, emergency department; *OR*, odds ratio; *CI*, confidence interval; *STONE*, Study of Tomography of Nephrolithiasis Evaluation.
