# Supplementary material for: Opioid Analgesic Use After an Acute Pain Visit: Evidence from a Urolithiasis Patient Cohort
Source: West J Emerg Med. 2022 Oct 23;23(6):864–71. doi: 10.5811/westjem.2022.8.56679 (PMC9683776; doi:10.5811/westjem.2022.8.56679)
Supplement: Supplementary file 1 [file wjem-23-864-s001.docx]

**Appendix Table 1.** Clinical and demographic characteristics of 1,296 participants with urolithiasis seen at 15 emergency departments from the STONE trial by missingness of reported pain at ED discharge.

|  | Total (n=1296) | Complete cases (n=892) | Missing reported pain at discharge (n=404) |
| --- | --- | --- | --- |
|  | n (%) | n (%) | n (%) |
| Gender |  |  |  |
| Female | 488 (37.7) | 343 (38.5) | 145 (35.9) |
| Male | 808 (62.4) | 549 (61.6) | 259 (64.1) |
| Age (years), median (IQR) | 39 (29–49) | 38 (28–48.5) | 41.5 (31–50) |
| Years of formal education |  |  |  |
| High school graduate or less | 601 (46.4) | 406 (45.5) | 195 (48.3) |
| Some post-high school education | 323 (24.9) | 232 (26.0) | 91 (22.5) |
| College graduate | 372 (28.7) | 254 (28.5) | 118 (29.2) |
| Race/ethnicity |  |  |  |
| Black or African American | 226 (17.4) | 139 (15.6) | 87 (21.5) |
| Hispanic | 317 (24.5) | 228 (25.6) | 89 (22.0) |
| Non-Hispanic white | 627 (48.4) | 437 (49.0) | 190 (47.0) |
| Mixed or other race | 126 (9.7) | 88 (9.9) | 38 (9.4) |
| Has health care insurance | 880 (67.9) | 651 (73.0) | 229 (56.7) |
| Pain at ED arrival |  |  |  |
| Low (0–3) | 62 (4.8) | 43 (4.8) | 19 (4.7) |
| Medium (4–8) | 492 (38.0) | 337 (37.8) | 155 (38.4) |
| High (9–10) | 742 (57.3) | 512 (57.4) | 230 (56.9) |
| Duration of pain before arrival |  |  |  |
| 1 to 2 hours | 316 (24.4) | 233 (26.1) | 83 (20.5) |
| 3 to 6 hours | 304 (23.5) | 228 (25.6) | 76 (18.8) |
| 7 to 12 hours | 155 (12.0) | 109 (12.2) | 46 (11.4) |
| 13 to 24 hours | 113 (8.7) | 72 (8.1) | 41 (10.2) |
| 25 to 48 hours | 98 (7.6) | 62 (7.0) | 36 (8.9) |
| > 48 hours | 310 (23.9) | 188 (21.1) | 122 (30.2) |
| Self-rated health |  |  |  |
| Excellent | 235 (18.1) | 162 (18.2) | 73 (18.1) |
| Very good | 363 (28.0) | 256 (28.7) | 107 (26.5) |
| Good | 457 (35.3) | 316 (35.4) | 141 (34.9) |
| Fair | 199 (15.4) | 132 (14.8) | 67 (16.6) |
| Poor | 42 (3.2) | 26 (2.9) | 16 (4.0) |
| State PDMP with online access | 668 (51.5) | 466 (52.2) | 202 (50.0) |
| Opioid analgesic during visit | 944 (72.8) | 661 (74.1) | 283 (70.1) |
| Opioid analgesic prescription at discharge | 1006 (77.6) | 710 (79.6) | 296 (73.3) |

Abbreviations: STONE, Study of Tomography Of Nephrolithiasis Evaluation; IQR, inter-quartile range; ED, emergency department; PDMP, prescription drug monitoring program.

**Appendix Table 2:** Clinical and demographic characteristics of 892 participants with urolithiasis seen at one of 15 emergency departments from the STONE trial by cohort.

|  | No opioid, no pain (n=72) | No opioid, pain (n=110) | Opioid,  no pain (n=210) | Opioid, pain (n=500) |
| --- | --- | --- | --- | --- |
|  | n (%) | n (%) | n (%) | n (%) |
| Gender |  |  |  |  |
| Female | 343 (38.5) | 24 (33.3) | 44 (40.0) | 66 (31.4) |
| Male | 549 (61.6) | 48 (66.7) | 66 (60.0) | 144 (68.6) |
| Age (years), median (IQR) | 38 (28–48.5) | 36 (28.5–46) | 36 (27–45) | 40 (30–51) |
| Years of formal education |  |  |  |  |
| High school graduate or less | 406 (45.5) | 44 (61.1) | 61 (55.5) | 79 (37.6) |
| Some post-high school education | 232 (26.0) | 13 (18.1) | 22 (20.0) | 60 (28.6) |
| College graduate | 254 (28.5) | 15 (20.8) | 27 (24.6) | 71 (33.8) |
| Race/ethnicity |  |  |  |  |
| Black or African American | 139 (15.6) | 10 (13.9) | 32 (29.1) | 26 (12.4) |
| Hispanic | 228 (25.6) | 31 (43.1) | 25 (22.7) | 60 (28.6) |
| Non-Hispanic white | 437 (49.0) | 23 (31.9) | 43 (39.1) | 100 (47.6) |
| Mixed or other race | 88 (9.9) | 8 (11.1) | 10 (9.1) | 24 (11.4) |
| Has health care insurance | 651 (73.0) | 45 (62.5) | 70 (63.6) | 167 (79.5) |
| Pain at ED arrival |  |  |  |  |
| Low (0–3) | 43 (4.8) | 3 (4.2) | 8 (7.3) | 19 (9.1) |
| Medium (4–8) | 337 (37.8) | 34 (47.2) | 40 (36.4) | 79 (37.6) |
| High (9–10) | 512 (57.4) | 35 (48.6) | 62 (56.4) | 112 (53.3) |
| Duration of pain before arrival to ED |  |  |  |  |
| 1 to 2 hours | 233 (26.1) | 20 (27.8) | 21 (19.1) | 64 (30.5) |
| 3 to 6 hours | 228 (25.6) | 19 (26.4) | 23 (20.9) | 51 (24.3) |
| 7 to 12 hours | 109 (12.2) | 8 (11.1) | 11 (10.0) | 29 (13.8) |
| 13 to 24 hours | 72 (8.1) | 3 (4.2) | 9 (8.2) | 16 (7.6) |
| 25 to 48 hours | 62 (7.0) | 6 (8.3) | 4 (3.6) | 16 (7.6) |
| > 48 hours | 188 (21.1) | 16 (22.2) | 42 (38.2) | 34 (16.2) |
| Self-rated health |  |  |  |  |
| Excellent | 162 (18.2) | 15 (20.8) | 14 (12.7) | 48 (22.9) |
| Very good | 256 (28.7) | 20 (27.8) | 25 (22.7) | 66 (31.4) |
| Good | 316 (35.4) | 28 (38.9) | 47 (42.7) | 68 (32.4) |
| Fair | 132 (14.8) | 8 (11.1) | 18 (16.4) | 23 (11.0) |
| Poor | 26 (2.9) | 1 (1.4) | 6 (5.5) | 5 (2.4) |
| ED visit in state with PDMP online access | 466 (52.2) | 22 (30.6) | 37 (33.6) | 116 (55.2) |
| Opioid analgesic administered during ED visit | 661 (74.1) | 31 (43.1) | 51 (46.4) | 157 (74.8) |
| Opioid analgesic prescription at ED discharge | 710 (79.6) | 0 (0.0) | 0 (0.0) | 210 (100.0) |
| Reported any pain at ED discharge | 610 (68.4) | 0 (0.0) | 110 (100.0) | 0 (0.0) |

Abbreviations: STONE, Study of Tomography Of Nephrolithiasis Evaluation; IQR, inter-quartile range; ED, emergency department; PDMP, prescription drug monitoring program.

**Appendix Table 3:** Sensitivity analysis: Adjusted odds ratios (95% CI) of using an opioid analgesic at each post-ED visit follow-up by reported pain at the end of the ED visit and receipt of prescription opioid analgesic from the ED in participants with suspected urolithiasis. Estimates from multivariable model weighted for propensity to receive an opioid analgesic prescription at ED discharge using multiple imputations with chained equations to account for missing outcome data at follow-ups. Sample includes 6,320 follow-up observations for 1,583 adults with suspected urolithiasis seen at one of 15 emergency departments in the STONE trial.

|  | No pain at discharge | Reported pain at discharge |
| --- | --- | --- |
|  | OR (95% CI) | OR (95% CI) |
| Opioid analgesic use at day 3 |  |  |
| No opioid prescribed at ED discharge | ref | ref |
| Opioid prescribed at discharge | 4.28 (2.54–7.20) | 3.87 (2.72–5.53) |
| Opioid analgesic use at day 7 |  |  |
| No opioid prescribed at ED discharge | ref | ref |
| Opioid prescribed at discharge | 2.56 (1.51–4.32) | 2.32 (1.57–3.44) |
| Opioid analgesic use at day 30 |  |  |
| No opioid prescribed at ED discharge | ref | ref |
| Opioid prescribed at discharge | 1.98 (1.12–3.50) | 1.79 (1.13–2.86) |
| Opioid analgesic use at day 90 |  |  |
| No opioid prescribed at ED discharge | ref | ref |
| Opioid prescribed at discharge | 1.87 (1.01–3.46) | 1.70 (0.98–2.94) |

Abbreviations: ED, emergency department; OR, odds ratio; CI, confidence interval; STONE, Study of Tomography Of Nephrolithiasis Evaluation.

**Appendix Table 4:** Unadjusted results from Table 2. Un-weighted odds ratios (95% CI) of using an opioid analgesic at each post-ED visit follow-up by reported pain at the end of the ED visit and receipt of prescription opioid analgesic from the ED. Estimates from multivariable model using multiple imputations with chained equations to account for missing outcome data at follow-ups. Sample includes 3,568 follow-up observations for 892 adults with urolithiasis seen at one of 15 emergency departments in the STONE trial.

|  | No pain at discharge | Reported pain at discharge |
| --- | --- | --- |
|  | OR (95% CI) | OR (95% CI) |
| Opioid analgesic use at day 3 |  |  |
| No opioid prescribed at ED discharge | ref | ref |
| Opioid prescribed at discharge | 3.87 (2.05–7.31) | 3.22 (2.02–5.13) |
| Opioid analgesic use at day 7 |  |  |
| No opioid prescribed at ED discharge | ref | ref |
| Opioid prescribed at discharge | 2.85 (1.41–5.77) | 2.37 (1.38–4.08) |
| Opioid analgesic use at day 30 |  |  |
| No opioid prescribed at ED discharge | ref | ref |
| Opioid prescribed at discharge | 2.05 (0.96–4.39) | 1.71 (0.88–3.31) |
| Opioid analgesic use at day 90 |  |  |
| No opioid prescribed at ED discharge | ref | ref |
| Opioid prescribed at discharge | 1.56 (0.58–4.15) | 1.30 (0.51–3.28) |

Abbreviations: ED, emergency department; OR, odds ratio; CI, confidence interval; STONE, Study of Tomography Of Nephrolithiasis Evaluation.

**Appendix Figure 1.** Proportion of participants reporting pain at follow-up visits by cohort estimated by multivariable model, stratified by pain reported and prescription of opioid analgesic at ED discharge.
